# Soil-to-plant transfer of ^40^K, ^238^U and ^232^Th and radiological risk assessment of selected mining sites in Nigeria

**DOI:** 10.1016/j.heliyon.2022.e11534

**Published:** 2022-11-11

**Authors:** Muyiwa Michael Orosun, Mojisola Rachael Usikalu, Kayode John Oyewumi, Maxwell Omeje, Gbonjubola Victoria Awolola, Oluwaseun Ajibola, Mark Tibbett

**Affiliations:** a Department of Physics, University of Ilorin, Ilorin, Nigeria; b Department of Physics, Covenant University, Ota, Ogun State, Nigeria; c Department of Industrial Chemistry, University of Ilorin, Ilorin, Nigeria; d Department of Applied Sciences, Federal College of Dental Technology and Therapy, Trans Ekulu, Enugu, Nigeria; e Department of Sustainable Land Management & Soil Research Centre, School of Agriculture Policy and Development, University of Reading, Whiteknights, Reading, RG6 6AR, United Kingdom

**Keywords:** Cancer, Radioactivity, Gamma spectroscopy, Risk assessment, Monte Carlo

## Abstract

One of the major route through which humans are exposed to ionizing radiation is via food chain, which is consequent of soil-to-plant transfer of radionuclides. This work reported the activity concentrations of ^40^K, ^238^U and ^232^Th in samples of water, soil and guinea corn grains collected from Beryllium and Gold mining sites in Kwara, Nigeria. In-situ measurements at approximately 1 m in the air was carried out using a well-calibrated portable Gamma Spectrometer (Super Spec RS-125), while the soil, water and the guinea corn samples were analyzed using a ‘3 × 3’ inch lead-shielded NaI (Tl) detector. The measured activity concentrations of the natural radionuclides in the soil from both mines are lower than the in-situ measurements. This was attributed to the contribution from other terrestrial materials on-site. The estimated mean transfer factors (TFs) for ^40^K, ^238^U and ^232^Th are 0.21, 0.17 and 0.31, and 0.46, 0.19 and 0.28 respectively for the Beryllium and Gold mining sites. While the TFs for ^238^U and ^232^Th exceeded the mean value of 0.0062 and 0.0021 for ^238^U and ^232^Th respectively, the TFs for ^40^K are well below the 0.74 for cereals grains provided by International Atomic Energy Agency (IAEA). The radiation impact assessment using the Monte Carlo simulations reveals values that were generally less than the global average values provided by the United Nations Scientific Committee on the Effects of Atomic Radiation (UNSCEAR). Hence, the risk of cancer inducement due to radiation exposure is within the acceptable limits for both mining sites.

## Highlights

• The measured activity concentrations of the primordial radioelements in the soil from both mines are lower than their corresponding *in-situ* measurements.
• Although the TFs for ^232^Th and ^238^U exceeded the average value of 0.0021 and 0.0062 for ^232^Th and ^238^U respectively, the TFs for ^40^K are below the 0.74 for cereals grains provided by International Atomic Energy Agency (IAEA).
• The excess lifetime cancer risks assessment using the Monte Carlo simulations reported values that are generally less than the global average values.

## Graphical abstract

**Figure:**
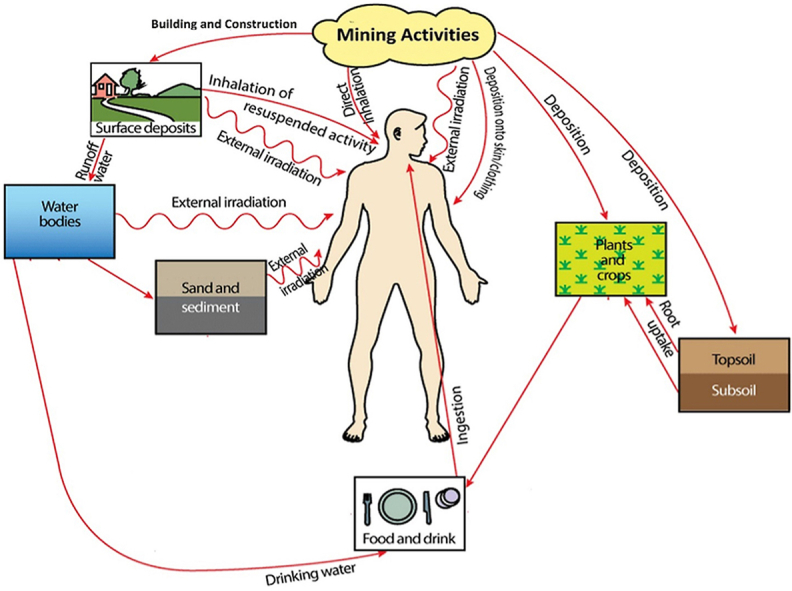

## 1 Introduction

Mining of tin, gold, beryllium, coal and other minerals has been associated with enhancement of naturally occurring radioactive materials in the environment because it involves the extraction of huge volumes of subsurface earths that usually contains varying levels of the naturally occurring radionuclides (Orosun et al., 2019). The health and ecological impacts of mining and dumping of the resulting tailings have been investigated by several scientists in Nigeria (Orosun et al., 2020a; Orosun et al., 2020b; Orosun et al., 2021; Orosun et al., 2022a; Adesiji and Ademola, 2019; Usikalu et al., 2019; Usikalu et al., 2022; Aliyu et al., 2015; Arogunjo et al., 2009; Ibeanu, 2002). The researchers measure the activity concentrations of the primordial radioelements in the mined mineral soils to evaluate the human health and environmental risks of the mining activities. The continuous mining of these mineral soils which contain radioactive materials could bring about the accumulation of radionuclides in soil, dust (air), water ways and feasibly taken up by crop plants, thus leading to extra redistribution through food chains (Aliyu et al., 2015). These radioactive elements disintegrate to give off hazardous radiations that are famous for causing several radiation induced health effects which could result in death (USEPA, 2018; Ajayi and Ajayi, 1999; Akinyose et al., 2018; Usikalu et al., 2018; Ajibola et al., 2022; Orosun et al., 2017; Orosun et al., 2018; Orosun et al., 2022b). According to the UNSCEAR (2000), IAEA (1996) and ICRP (1991) reports, the global average values in soils for background exposures to ^40^K, ^238^U and ^232^Th are given as 420.00, 32.00, and 45.00 Bqkg^−1^ respectively.

Reports can be found in literatures on radioactivity in soil, water and foods from different countries. In Nigeria for instance, many researchers have assessed the impact of mining activities on the human health and the ecosystem in some parts of the country. Table 1 provides the results of these studies. Most of these studies were carried out in the suburbs of Kogi, Nassarawa and Jos in central part Nigeria where mining activities are prevalent. The health effects due to natural radioactivity because of the enhancement of these radioelements in soils from mining regions of Nasarawa was investigated by Aliyu and his team in 2015. They associated the observed higher dose rates with the mining activities in the areas. They concluded that in all the cases they considered, the risks are not likely to be grievous. Similarly, the activity concentrations of ^232^Th, ^40^K, and ^238^U in resulting tailings from coal mining location in Enugu was presented by Balogun et al. (2003). Also, Jibiri and Esen (2011) measured the activities of these primordial radioelements in groundwater within uranium mineralized regions in Nigeria. Arabi and his team of researchers, in 2013 resolved that the contamination of groundwater by the primordial radioelements in the neighborhood of uranium mine is evident. They postulated that the water serves as a means of conveying and increasing uranium in waterways of locations far away from the mines. Although, they concluded that the level of radiation hazard may not cause any substantial or severe health effects (Arabi et al., 2013).

**Table 1:** Results of radioactivity measurements of ^232^Th, ^40^K and ^238^U of some selected studies around the world.

Case Study	^238^U (Bqkg^−1^)	^232^Th (Bqkg^−1^)	^40^K (Bqkg^−1^)	Country	References
Kaolin	38.20	65.10	93.90	Nigeria (Ifonyintedo)	Adagunodo et al. (2018)
Tin Mine	3779.10	8175.20	-	Nigeria	Ibeanu (2002)
Kaolin (soil)	82.00	94.80	463.60	Turkey	Turhan (2009)
Clay (soil)	39.30	49.60	569.50	Turkey	Turhan (2009)
Floor ceramic	101.22	87.53	304.57	Iraq	Amana (2017)
Wall ceramic	102.12	70.90	328.60	Iraq	Amana (2017)
Kaolin (soil)	964.70	251.60	58.90	Eqypt	El-Dine et al. (2004)
Phosphogypsum	206.80	99.10	15.10	Brazil	Mazzilli and Saueia (1999)
Coal mine	62.78	54.74	82.43	Enugu, Nigeria	Berekta and Mathew (1985)
Sands (soil)	78.00	33.00	337.00	Egypt	El-Afifi et al. (2006)
Granite (In-situ)	18.15	42.86	570.91	Nigeria	Orosun et al. (2019)
Soil and Rock	13.60	24.20	162.10	Ghana	Faanu et al. (2011a)
Laterite (soil)	30.00	41.00	65.00	Nigeria (Obajana)	Ajayi et al. (2012)
Granite (Soil)	11.51	15.42	441.06	Nigeria	Orosun et al. (2020a)
Soil Samples	55.30	26.40	505.10	Nigeria	Ademola et al. (2014)
Soil and Rock	32.00	45.00	420.00	Global Limit	UNSCEAR (2000)

Tailings from mining activities are often dumped in the region of the mining sites in tons. This exploration and the throwing away of tailings containing enhanced level of radionuclides causing serious human health effects. Despites the massive mining activities that is going on in this part of the country, no research work had been carried out on the radio-ecological impacts of the mining activities until our earlier work (Orosun et al., 2020c) where *in-situ* radioactivity measurements of ^238^U, ^40^K, and ^232^Th was conducted using handheld RS-125 gamma-spec. The on-field measurements revealed enhanced levels of these primordial radionuclides. This therefore demands further research on food crops and drinking water sources using higher resolution laboratory based NaI (Tl) detector. Thus, baseline study that relied on universally approved methodology regarding human health implications of mining in Kwara is apposite. Therefore, this study is aimed at carrying out radioactivity measurements of ^238^U, ^232^Th and ^40^K in samples of and guinea corn grains, soils and water collected from gold and beryllium mining sites in Kwara, Nigeria and use the results to evaluate the human health effects associated with the mining activities using the radiological hazard parameters and Monte Carlo Simulations. The Government and other authorities relies on the outcome of independent research like this to regulate the indiscriminate mining activities.

## 2 Materials and methods

### 2.1 Study area

**Figure 1:**
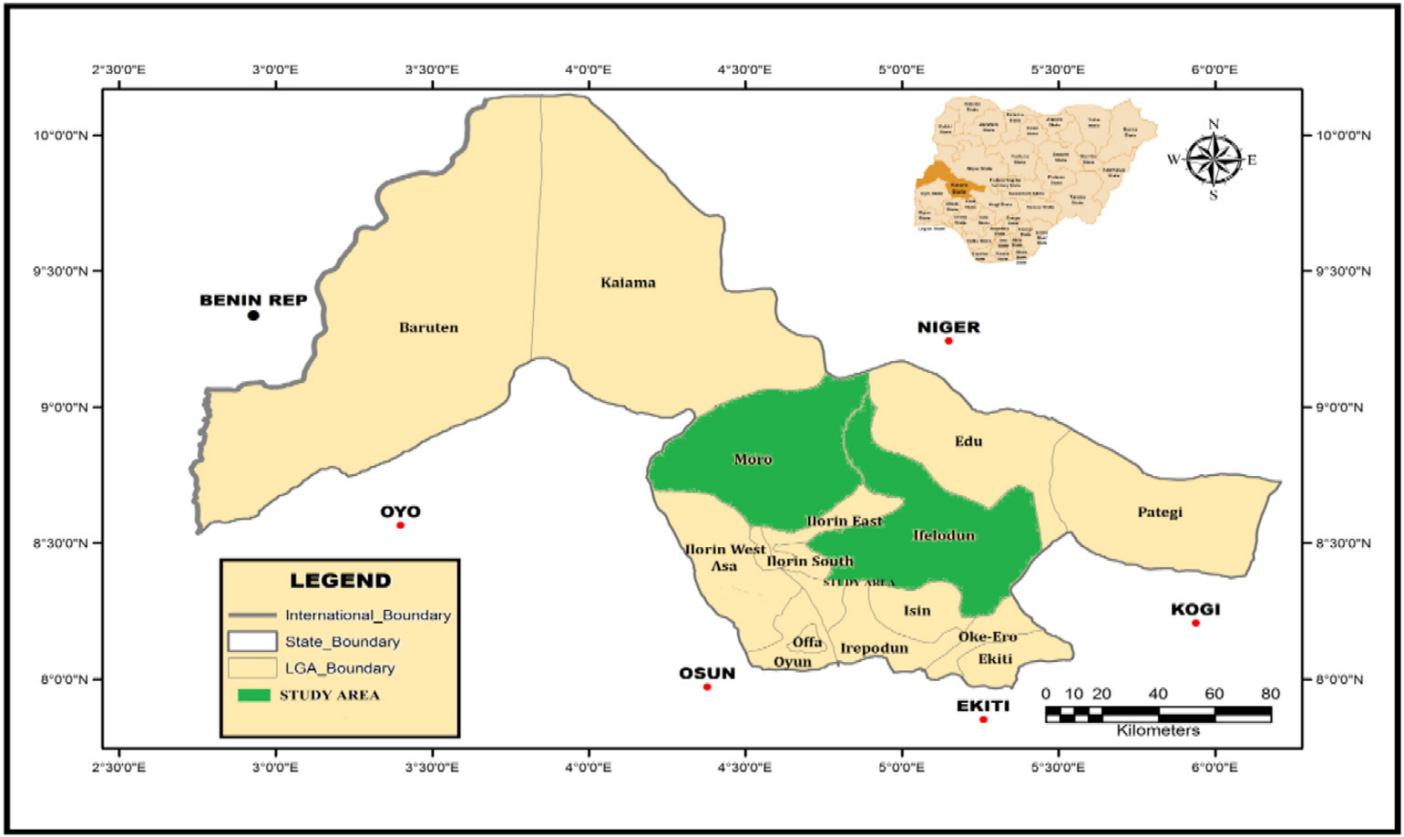
Map of Nigeria showing the study areas.

Ifelodun and Moro are Local Government Areas (LGA) of Kwara State, Nigeria, located between Longitudes 4°25′E and 4°65′E and latitudes 8°20′N and 8°50′N (Figure 1) (Orosun et al., 2020c). While Ifelodun is the biggest LGA in Kwara, covering 3,435 km^2^ and has 206,042 people as recorded in the 2006 census. Moro with a landmass of 3272 km^2^ has a total population of 108,792 people (City population, 2016). Kwara state has an elevation of 251 m (823 feet) with a humid wet-dry climate and an average yearly rainfall of 1,200 mm (Orosun et al., 2020b). The yearly average temperature of Kwara is 26.2 °C, with peak temperature of around 30 °C during the hottest month, March. In this part of the country, wet season is between April and October, while dry season is between November and March. The geology of this research region is of crystalline pre-Cambrian basement complex rocks that are basically igneous and metamorphic form the soil. The metamorphic rock comprises biotite gnesiss, granitic gnesiss, quartzite augitegnesiss and banded gnesiss. The intrusive rock comprises of pegmatite and vein quartz (Kayode et al., 2015; Ajadi et al., 2016; Oyegun, 1985).

In Kwara state, gneisses and schist are usually related to gold mineralization (Orosun et al., 2020c). Around the Nupe basin, and the basement complex of Bishewa and Gidan sani in Moro, sandstones are identified as gold host. Assessment of these potentials in Panned concentrate of rivers and soil sediments in Bishewa, koro, Gidan sani and river Wara yielded around 4.1 g/t–8.3 g/t, nevertheless, an asses of nearly all of the gneisses around these coverage varies between 1.1 g/t and 4.5 g/t. Ndanaku, Mari, Oputa, Lokomosi, Tunga Bichi, Gbajubo, Giloadi and Birnin Ruwa in Edu and Ifelodun LGA areas discovered. In Ndanaku and Birnin Ruwa, preliminary results yielded an average of 5 g/t for gold. The implication is that the area of mining activities and mineralization is extensive.

### 2.2 *In situ* radioactivity measurements using portable RS125 gamma spectrometer

The super SPEC RS-125 detector with 2.0 × 2.0 NAI crystal was used to measure the *in situ* activities of ^40^K, ^232^Th, ^238^U and the doses of radiation exposures at about 1 m above the ground. The portable RS125 gamma spectrometer was made by Canadian geophysical institute Canada. The detector is made up of 2.0 × 2.0 NaI crystal. The calibration was carried out on a 1 × 1 m test pads with the use of 5 min spectral accumulation for uranium, potassium and thorium for 10 min for terrestrials’ pads. The portable gamma spectrometer has a wide-ranging energy of 30–3000 keV capable of detecting radiation of terrestrial origin. More information about RS125 gamma spectrometer can found in previous studies where the instrument was used (Orosun et al., 2020b, 2020c; Adagunodo et al., 2018). Measured values available in our article data (Orosun et al., 2020c).

### 2.3 Sample preparation

Samples of topsoil and guinea-corn grains within the same rooting location were obtained from each of the mining sites during the periods of harvesting in November 2019. Forty-eight (48) samples (24 samples each) of topsoil from the Beryllium and Gold mining fields were obtained within the rooting zones of the guinea-corn plant marked for sampling. The collected soil samples were labelled and sent to the laboratory for the removal of traces of plastics, glasses, animal and plant matter. With this, there is a guarantee that the samples undergoing the analysis are not littered with impurities of such kind. Agate mortar was used to pulverised the samples, put through a 1 mm mesh and then reserved in cylindrical plastic Marinelli beakers taped up by means of adhesive tape to stop radon gas from escaping. The labeled samples were preserved for forty (40) days after which the gamma-ray spectroscopy was performed in order to attain secular radioactive equilibrium. Forty-eight (48) samples of guinea corn grains (24 samples each) and 24 water samples (12 samples each) were also obtained from the investigating mining zones. An automatic blending machine was used to grind the guinea-corn grain samples into powdery form after the samples were oven dried at 70 °C (Ugbede and Osahon, 2021) until constant weight was achieved. The water samples were collected, measured using digital balance of detection limit of 0.01 g, subjected to heat under the temperature of about 60 °C to concentrate the samples for a better detection of activity level of the targeted radionuclides (Maxwell et al., 2015). At the collection site, the water samples were filtered to remove undesirable solid particles and other suspended particles. The water samples were acidified with 11 M HCl at the rate of 10 ml per liter instantaneously after collection to ensure prevention of absorption of the radioelements of interest into the walls of the containers. Aliquots of 500 ml of each of the filtered and heated water sample was measured into the Marinelli beakers. The plastic test containers (Marinelli cylindrical beakers) were used to keep the powdered guinea corn and water samples. The containers were thoroughly washed with liquid detergents and oven dried before use (Faanu et al., 2011b). All the water and grain samples were kept in cylindrical plastic containers of uniform geometry, taped via adhesive tape to prevent Radon gas from escaping. The samples were also stored up to 40 days so that secular radioactive equilibrium is guaranteed before the gamma-ray spectroscopy (Orosun et al., 2020a).

### 2.4 Gamma-ray spectrometry

The detector used for radioactivity measurement was a 3 × 3 inch lead – shielded NAI (TI) detector. Princeton Gamma Tech. USA manufactures the NAI (TI) detector. It is connected to multichannel analyzer (MCA) (GS-2000-Pro) by means of a pre-amplifier. The RSS8 gamma source set (from spectrum techniques LLC, USA) was used to calibrate the energy of the NAI (TI) detector. The calibration procedures were as outlined by Orosun et al. (2020a) and Faanu et al. (2011b).

**Figure 2:**
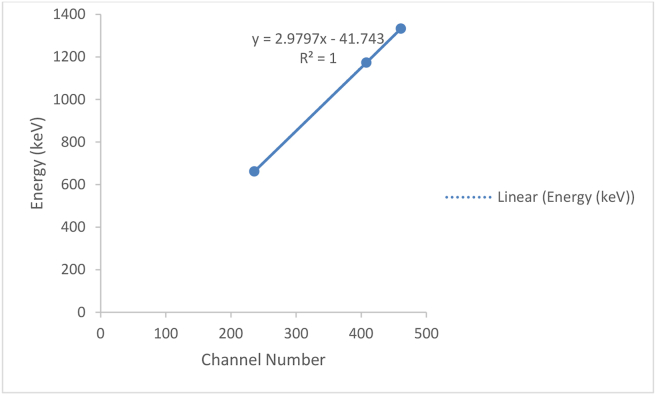
Energy calibration curve.

Table 2 gives the channel number of each point source energy level registered in spectrometry system. Figure 2 present the energy calibration curve, the linear relationship observed showed that the spectrometry system experiences minimal error at the peak of each source energy.

**Table 2:** Energy calibration data.

Source	Channel Number	Energy (keV)
^137^Cs	236	662
^60^Co	408	1173
^60^Co	461	1332

The full energy peak efficiency was utilized as it links the peak area in the spectrum to the quantity of radioactivity present. It is given by Eq. (1).

(1) $$\varepsilon =\frac{C_{net}}{A\times P_{\gamma }\times T}$$

Here, A represents the source radionuclide activity, Pγ represents the measured absolute gamma ray emission probability of the radionuclide, T stands for acquisition time and C_net_ represents the net peak count of the source's radionuclide. The detection efficiency calibration was carried out using standard reference materials IAEA-RG set (IAEA-RGK-1, IAEA-RGU-1, and IAEA-RGTh-1) for the soil, IAEA-445 for water and IAEA-464 for guinea-corn consisting of known activities of ^40^K, ^238^U and ^232^Th. An empty container was used to obtain the background gamma count by taking count of it for 18000 s prior to the sample measurement. After attaining the secular equilibrium, all the samples were positioned and counted accordingly on the detector in the same 18000 s. As outlined by Orosun et al. (2020a), 2614.7 keV of ^208^Tl and 911.21 keV of ^228^Ac were used in estimating ^232^Th activities, the activities of ^214^Bi (from its 609.31 and 1764.5 keV γ-ray peak) and ^214^Pb (295.21 and 351.92 keV) were used as indicators for ^238^U in the samples. The activity concentration of ^40^K was obtained from the 1460 keV γ-rays released for the duration of its disintegration process. The LLD and MDA was estimated using the peak of ^137^Cs that occurs at 662 keV and the peak of ^40^K that occurs at 1460.83 in the background spectrum (Cember, 1996).

Eq. (2) below was used to determine the activity concentration A measured in Bqkg^−1^ or Bql^−1^, of each radioelement of interest for all samples.

(2) $$A=\frac{Cnet}{\gamma \times \varepsilon \left(E\gamma \right)\times T\times Ms}$$

where, C_net_ represents the net count rate, γ is the absolute intensity of the gamma-ray, ε(E_γ_) is the detector efficiency at the specific gamma ray energy (E_γ_), t is the full counting time (18,000 s), and M_s_ is the mass of the given sample in kg.

### 2.5 Radiological hazard evaluation

#### 2.5.1 Absorbed dose rate

Above the soil at 1 m high, the outdoor dose rate was taken *in situ* with the use of RS-125 gamma spec and in air, the absorbed dose rate was measured with respect to the mean specific activities of ^232^Th, ^40^K and ^238^U (Bqkg^−1^) in the soil. This was estimated using Eq. (3) below.

(3) $$D\;\left({nGyh}^{-1}\right)=\left(0.0417\right)CK+\left(0.462\right)CU+\left(0.623\right)CTh$$

where CTh, CK and CU are the activities of ^232^Th, ^40^K, and ^238^U respectively in the soil samples (UNSCEAR, 2000).

#### 2.5.2 Annual effective dose (AED_External exposure_)

The dose rates were used to evaluate the annual effective dose absorbed by a member of the general public due to the levels of ^40^K, ^238^U and ^*2*32^Th (Bqkg^−1^), with an occupancy factor of 0.2 for outdoor exposure and dose conversion factor of 0.7 SvGy^−1^ (UNSCEAR, 2000; Isinkaye et al., 2015).

(4) AED_External_ (mSvy^−1^) = D (nGy h^−1^) × 8760 h × 0.7 (SvGy^−1^) × 0.2 × 10^−6^

(5) AED_External-insitu_ (mSvy^−1^) = DR (nGy h^−1^) × 8760 h × 0.7 (SvGy^−1^) × 0.2 × 10^−6^

#### 2.5.3 Representative level index (RLI)

The Representative Level Index was appraised using Eq. (6) below (UNSCEAR, 2000):

(6) $$RLI=\frac{C_{u}}{150}+\frac{C_{Th}}{100}+\frac{C_{k}}{1500}\leq 1$$

where all the parameters maintain their usual meanings.

RLI values = 1 corresponds to an AED ≤ 1 mSv. Hence, the representative level index is an index for selecting environmental parameters containing substantial quantities of these natural radioelements and evaluate the health risk associated with them (UNSCEAR, 2000).

#### 2.5.4 Annual effective dose for ingested radioelements (AED_Internal_)

The AED_Internal_ attributable to consumed radioelements in the water samples and the grain (guinea corn) was evaluated using Eq. (7)

(7) $${AED}_{Internal}=365\sum_{i}I_{i}\times D_{i}$$

where *I*_*i*_ represents daily intake of the primordial radionuclides due to ingestion of the food or water (Bqd^−1^) = (concentration of radionuclides in foodstuff or water) × (food or water ingestion rate) and D_i_ represents the dose factor. The dose coefficient (D_i_) for ^40^K, ^238^U and ^234^Th are: 6.2 × 10^−9^, 4.5 × 10^−8^ and 2.3 × 10^−7^ SvBq^−1^, respectively (UNSCEAR, 2000; ICRP, 2012). 2 ld^−1^ was adopted as the intake of water on daily basis. Nigerian per capita annual consumption of guinea is about 24.8 kg (Orosun et al., 2020a).

#### 2.5.5 Excess lifetime cancer risk (ELCR**)**

The *ELCR* was evaluated using Eq. (8):

(8) ELCR = AED × DL × RF

where, AED is the Annual Effective Dose for either external or internal exposures provided by Eqs. (4), (5), and (7), DL represents the mean life time (assuming seventy years as the life expectancy of an average Nigerian) and RF stands or the Stochastic Risk Factor (Sv^−1^), i.e. fatal cancer risk per Sievert. To obtain the stochastic effects for the general public, ICRP uses RF as 0.05 (UNSCEAR, 2000). The recommended limit is 3.75 × 10^−3^ for internal exposure, 0.29 × 10^−3^ for outdoor external exposure and 1.16 × 10^−3^ for indoor external exposure (UNSCEAR, 2000).

### 2.6 Monte Carlo simulation (MCS) for the ELCR

Regarding the environmental protection's perspectives on the analysis of risk, decision-making vis-à-vis needs for mitigation or remediation is a crucial task. Scientists usually encounter uncertainties and obscurities in risk analysis. While overestimation of the excess lifetime cancer risk (*ELCR*) can lead to waste of scarce resources on needless remediation, its underestimation is risky because it is capable of giving rise to preventable health consequences among dwellers. Assessing the lifetime cancer risk using the risk model presented by Eq. (8) either underestimates or overestimates the actual cancer risk. Therefore, this study uses probabilistic approach by means of the Monte Carlo simulation (*MCS*) so as to robustly evaluate further pragmatic risks associated with those primordial radionuclides (^238^U, ^232^Th and ^40^K) present in the environmental parameters.

The MCS probabilistic approach has the greater advantage of exposing scientists to all the feasible outcomes in order to evaluate the impact of risk, so as to make better decisions under uncertainty. The Monte Carlo simulation (*MCS*) performs the risk analysis by developing models that produces all possible outcomes through interchanging array of values (probability distribution) for factors containing intrinsic uncertainty (Omeje et al., 2022; Shahrbabki et al., 2018; Karami et al., 2019). The MCS then computes the outcomes severally (with ten thousand trials in this work), expending several random values from the probability functions in each occasion. Generally, the initial step of the Monte Carlo simulation involves defining the hypotheses and provide the probability distribution types and parameters of the input variables of the model. The indeterminate variables with intrinsic uncertainties include the results of the radioactivity measurements, the adopted occupancy values, risk factor and the lifetime duration. The subsequent step involves defining the prediction variables in accordance to the model for the cancer risk, and then state the number of iterations (10000 in this case) and begin the simulation. The concluding aspect involves interpretation of the uncertainty of the predicted variables in accordance with the operation results (Usikalu et al., 2022a). The Monte Carlo Simulations was carried out using Oracle Crystal Ball software version 11.1.2.4.850.

### 2.7 Bioaccumulation/transfer factor (TF)

The ratio of the concentrations of the primordial radionuclide present in the grain to that of the corresponding soil is commonly called the Bioaccumulation Factor or Transfer Factor (TF). The transfer factor (TF) for food according to IAEA (1989) is defined as:

(9) $$TF=\frac{Activity\;Concentration\;in\;Food\frac{crop}{plant}\left(dry\;weight\right)\;\left(\frac{Bq}{kg}\right)}{Activity\;Concentration\;in\;Soil\;\left(dry\;weight\right)\;\left(\frac{Bq}{kg}\right)}=\frac{C_{guinea-corn}}{C_{soil}}$$

where $C_{guinea-corn}$ and $C_{soil}$ denotes the corresponding radionuclide activity (i.e. ^40^K, ^238^U, and ^232^Th) present in the guinea corn samples and soils respectively. One of the main way in which human are exposed to ionizing radiation through food chain is Soil-to plant-transfer. In a situation where TF is less than one, it means that the radionuclide is only absorbed by the guinea corn but not accumulated alternatively, TF greater than one implies that the radionuclide is accumulated by the plant.

## 3 Results and discussion

### 3.1 In situ gamma dose rate (DR) and activity concentration of ^238^U, ^232^Th and ^40^K

The *in situ* measured gamma dose rate (*DR*) and the activity concentrations of ^238^U, ^232^Th, ^40^K and together with their statistical summaries for the Gold mining field in Moro and Beryllium mining field in Ifelodun are obtainable via the data article Orosun et al. (2020c). Tables 3 and 4 presents the result's summary. From Table 3, the lowest values of the activity concentration of ^40^K, ^238^U, ^232^Th and DR for the Beryllium mining field in Ifelodun LGA are 93.90, 1.24, 5.68 Bqkg^−1^ and 19.10 nGyh^−1^, while their respective highest values are 1001.60, 65.46, 41.01 Bqkg^−1^ and 63.90 nGyh^−1^. From Table 4, the lowest values of the radioactivity measurements of ^232^Th, ^40^K, ^238^U, and DR for the Gold mining field in Moro LGA are respectively 11.37, 406.90, 3.71 Bqkg^−1^ and 56.30 nGyh^−1^, and their respective highest values are 970.30, 97.57, 73.89 Bqkg^−1^ and 90.30 nGyh^−1^. The measured mean activities of ^40^K, ^238^U, ^232^Th and DR for the Gold mining field were found to be respectively; 647.74, 43.47, 45.61 Bqkg^−1^ and 77.11 nGyh^-^. While the measured mean activities of ^40^K, ^238^U, ^232^Th and DR for the Beryllium mining field are 412.99, 19.83, 20.62 Bqkg^−1^ and 39.53 nGyh^−1^, correspondingly. The average values of ^238^U, ^40^K, ^232^Th and DR for the Gold mine field exceeds that of the estimated averages for Beryllium mining site.

**Table 3:** Descriptive Statistics of the in-situ radioactivity measurements of ^40^K, ^238^U, ^232^Th and Dose Rate of Beryllium mining site.

	^238^U (Bqkg^−1^)	^40^K (Bqkg^−1^)	^232^Th (Bqkg^−1^)	DR (nGyh^−1^)
Min–Max	1.24–65.46	93.90–1001.60	5.68–41.01	19.10–63.90
Mean	19.83	412.99	20.62	39.53
SD	17.09	250.83	10.62	11.80
CV	86.20	60.73	51.51	29.86
Skewness	0.79	0.89	0.27	0.15
Kurtosis	−0.14	−0.33	−0.92	−0.88
Global average	32.00	420.00	45.00	59.00

**Table 4:** Descriptive Statistics of the *in-situ* radioactivity measurements of ^40^K, ^238^U, ^232^Th and Dose Rate of Gold mining site.

	DR (nGyh^−1^)	^40^K (Bqkg^−1^)	^238^U (Bqkg^−1^)	^232^Th (Bqkg^−1^)
Min–Max	56.30–90.30	406.90–970.30	3.71–97.57	11.37–73.89
Mean	77.11	647.74	43.47	45.61
SD	7.87	152.94	23.89	13.91
CV	10.21	23.61	54.97	30.49
Skewness	−0.36	0.48	0.16	−0.33
Kurtosis	0.11	−0.50	−0.74	0.01
Global average	59.00	420.00	32.00	45.00

According to the recommendations from ICRP (1991), IAEA (1996) and UNSCEAR (2000), the global average values for background exposure to, ^232^Th, ^40^K, ^238^U and DR are 45.00, 420.00, 32.00 Bqkg^−1^ and 59.00 nGyh^−1^, respectively. It was discovered that, while the entire estimated average values of ^238^U, ^40^K, ^232^Th and Dose Rate for the Beryllium minefield in Ifelodun falls in the harmless limits recommended. All average values for the Gold mining field in Moro were higher than the recommended global averages. These high values observed at Moro, calls for serious worry because significant elevation in the level of the natural radionuclides can cause rise in the background radiation level, leading to exposure to elevated radiation levels.

### 3.2 Radioactivity content in the selected soil samples using laboratory based “3 × 3” inch lead-shielded NaI(Tl) detector

Since *in situ* measurements are not enough to stand for quantitative determination of specific activity concentrations, we have decided to consolidate our measurements with laboratory analyses. Tables 5 and 6 presents the statistical summary of the gamma spectroscopy of Beryllium mining fields and gold mining fields in Ifelodun and moro respectively, using the laboratory based 3 × 3 inch lead-shielded NAI (TI) detector. The regular radionuclide as expected belonged to the decay series led by ^238^U and ^232^Th alongside non-series ^40^K. The activities of ^40^K dominated the activities of ^238^U and ^232^Th throughout the measured areas all the locations. The values obtained for all the radionuclide (^238^U, ^232^Th and ^40^K) for both mining zones were moderately skewed.

**Table 5:** Descriptive Statistics of the Activities of ^232^Th, ^40^K, ^238^U in the soil samples obtained from Beryllium mining site.

Sample location	Sample stat	^238^U (Bqkg^−1^)	^40^K (Bqkg^−1^)	^232^Th (Bqkg^−1^)
Ifelodun	Min–Max	3.51–18.94	120.19–810.88	5.29–24.29
(Beryllium)	Skew	−1.48	−1.05	−1.43
Soil	Kurt	−0.14	−0.33	−0.92
	Mean ± SD	12.82 ± 8.19	509.74 ± 353.73	16.63 ± 10.02
	Global average	32.00	420.00	45.00

**Table 6:** Descriptive Statistics of the Activities of ^232^Th, ^40^K, ^238^U in the soil samples obtained from Gold mining site.

Sample location	Sample stat	^238^U (Bqkg^−1^)	^40^K (Bqkg^−1^)	^232^Th (Bqkg^−1^)
Moro	Min–Max	10.72–11.73	327.38–405.14	13.78–14.35
(Gold)	Skew	−0.03	−1.05	−0.85
Soil	Kurt	−0.74	−0.5	0.01
	Mean ± SD	11.73 ± 0.51	371.23 ± 39.82	14.35 ± 0.29
	Global average	32.00	420.00	45.00

The measured mean activities of ^*40*^K, ^238^U as well as ^232^Th in the Beryllium mining field were found to be 509.74, 12.82 and 16.63 Bqkg^−1^ respectively and for Gold mining field we have 371.23, 11.73 and 14.35 Bqkg^−1^ respectively. The measured mean values of the radionuclide for the Beryllium mining field were surprisingly slightly higher than that of the estimated mean values for Gold mining field. This is not in agreement with the *in situ* measurements where reverse was the case. Interestingly, all the activity concentrations of ^40^K, ^238^U and ^232^Th measured throughout all the areas are found to be lesser than their corresponding *in situ* measurements. The contributions of earth materials to gamma ray detection for the *in situ* measurements were believed to be the reason for elevated concentration of those radionuclides. This suggests that miners have greater risk of exposure to ionizing radiations compared to residents who used the soils for building and other purposes.

The measured mean values of the primordial radionuclides and the dose rates for both mine fields were compared with those in some selected published knowledge across the world as shown in Table 1. The results shows that the mean values obtained in this study concurred well with the values reported for Ifonyintedo (Oyo State, Nigeria) by Adagunodo et al. (2018) and granite mine field results in Asa (Kwara state) by Orosun et al. (2019 and 2020a).

### 3.3 Radioactivity content in the water and grain samples using laboratory based “3 × 3” inch lead-shielded NaI(Tl) detector and the transfer factor (TF)

For a holistic study, one must consider exposure through water intake and food chain having confirmed the activities of the primordial radionuclides in the soil. The food crop commonly grown and consumed in these regions is guinea corn. This is chiefly due to the ability of guinea corn to survive drought and thrive in arid terrain. Guinea corn is used in preparing certain yummy meals such as the renowned African porridge as well as pancakes. Tables 7, 8, 9, and 10 show the results of the of the gamma spectrometry analysis of guinea corn and water samples collected from both mining fields.

**Table 7:** Activities of ^*2*32^Th, ^40^K and ^238^U in the water samples obtained from Beryllium mining site.

Sample location	Sample stat	^238^U (Bqkg^−1^)	^40^K (Bqkg^−1^)	^232^Th (Bqkg^−1^)
	MIN	0.35	11.27	0.51
Ifelodun	Max	1.06	30.67	1.3
(Beryllium)	Mean ± sd	0.80 ± 0.39	23.65 ± 10.75	1.03 ± 0.45
	Global average	1.00	10.00	1.00

**Table 8:** Activities of ^232^Th, ^40^K and ^238^U in the water samples obtained from Gold mining sites.

Sample location	Sample stat	^238^U (Bql^−1^)	^40^K (Bql^−1^)	^232^Th (Bql^−1^)
	Min	0.83	25.93	1.16
Moro water	Max	1.21	35.87	1.56
(Gold & lead)	Mean ± sd	1.00 ± 0.19	29.86 ± 5.29	1.30 ± 0.23
	Global average	1.00	10.00	1.00

**Table 9:** Activities of ^232^Th, ^40^K and ^238^U in the sampled guinea corn grains obtained from Beryllium mining sites.

Sample location	Sample stat	^238^U (Bqkg^−1^)	^40^K (Bqkg^−1^)	^232^Th (Bqkg^−1^)
	Min	1.72	97.48	5.17
Ifelodun grains	Max	2.43	110.03	5.25
(Beryllium)	Mean ± sd	2.19 ± 0.41	105.85 ± 7.25	5.22 ± 0.05
	TF	0.17	0.21	0.31

**Table 10:** Activities of ^232^Th, ^40^K and ^238^U in the sampled guinea corn grains obtained from Gold mining sites.

Sample location	Sample stat	^238^U (Bqkg^−1^)	^40^K (Bqkg^−1^)	^232^Th (Bqkg^−1^)
	Min	0.18	24.39	2.39
Moro grains	Max	4.7	257.23	7.39
(Gold & lead)	Mean ± sd	2.19 ± 1.30	179.62 ± 134.43	4.08 ± 2.87
	TF	0.19	0.46	0.28

The estimated mean values of the activities concentration of ^40^K, ^238^U and ^232^Th in the water samples are 23.65, 0.80 and 0.51 Bql^−1^ respectively for the Beryllium mining site and 29.86, 1.00 and 1.30 Bql^−1^ respectively for the Gold mining site (see Tables 7 and 8). For the guinea corn samples, the estimated mean values of the activities concentration of ^40^K, ^238^U and ^232^Th are 105.85, 2.19, 5.22 and 179.62, 2.19, 4.08 Bqkg^−1^ respectively for the Beryllium and Gold mining sites (see Tables 9 and 10). The mean activities of ^40^K in the water and guinea corn samples were higher than the ^238^U and ^232^Th activities in all the locations. While the mean values of all the primordial radionuclides in water samples from the Gold mining are higher than the ones recorded at Beryllium mining, the mean values in the guinea corn samples are only higher for ^40^K. The presence of these primordial radionuclides in high concentration in the grains is worrisome since their high activities can lead to internal exposure that is very harmful to human health. Remember one of the prominent ways of exposure to ionizing radiation in human is through food chain.

#### 3.3.1 Soil-to-plant transfer factors of radionuclides

The transfer/bioaccumulation factor (TF), which was estimated as the ratio of the mean activities of ^40^K, ^238^U and ^232^Th in the soil to their respective mean activities in the guinea corn are 0.21, 0.17 and 0.31 respectively for the Beryllium mining, and 0.46, 0.19 and 0.28 respectively for the Gold mining. These values for the bioaccumulation factor exceeded the mean value of 0.0062 for ^238^U and 0.0021 for ^232^Th, for cereals grains provided by IAEA (2010) handbook of parameter values for the prediction of radionuclide transfer factors. However, the values reported in this study for ^40^K (i.e. 0.21 and 0.46 for the beryllium mining and gold mining fields respectively) are well below the 0.74 for cereals grains provided by IAEA (2010) handbook of parameter values for the prediction of radionuclide transfer factors. Nevertheless, these values for the bioaccumulation factor are very well comparable with the findings of Orosun et al., (2020a) where they reported the mean transfer factors as 0.49, 0.46 and 0.58 respectively for ^40^K, ^238^U and ^232^Th, for guinea-corn grains cultivated around granite mining field in Asa, North-central, Nigeria. In broader sense, the estimated transfer factors from this research work are within the range of values reported by Oluyide et al. (2018) for maize, cassava and different varieties of vegetables; Avwiri and Agbalagba (2013) for cocoyam, yam and cassava harvested in the vicinity of a fertilizer factory in Onne, Nigeria; and the soil-to-plant transfer factors for rice from Ezillo paddy fields in Ebonyi State, Nigeria reported by Ugbede and Osahon (2021).

By and large, the values of the transfer factors obtained in this study portrays the transmission/mobility of the primordial radionuclides from the soil via the rooting system to the grains. Although natural properties of the soil like the conductivity, chemical substances present, pH and soil texture/type, along with the mobility and solubility of the radioelements have been reported as factors affecting the absorption and transmission/mobility of the radioelements in plants (Ugbede and Osahon, 2021).

### 3.4 Results of the estimated radiation hazard parameters

In order to assess the level of radiological impacts related to the use of the soil and the ingestion of guinea corn and water from both mining fields, the radiation hazard indices were calculated using the measured activity concentration of the natural radionuclides. Just as expected, the estimated mean values of the impact parameters for the *in situ* measurements are higher than the impact parameters for the Lab measurements for the soil samples for both mining sites. While the (*in situ* and Lab) impact parameters for the Beryllium mining are within the acceptable limits provided by UNSCEAR, the radiological impact parameters for the *in situ* measurements for the Gold mining exceeds the limits given UNSCEAR (see Table 11). It implied that the risk of exposure to gamma radiation is relatively higher at the Gold mining site. Even though these values are lower at the Beryllium mining site, the inhabitants may not be safe from radiation related health effects because all ionizing radiations are harmful for stochastic effects. The result also showed that all the averages of the hazard parameters due to the ingestion of these radionuclides in the water and the guinea corn are less than the recommended values (Table 12).

**Table 11:** Radiation hazard indices for the sampled soils.

Location		D (nGyh^−1^)	AED_outdoor_ (mSvy^−1^)	RLI	ELCR (X 10^−3^)
Ifelodun soil	(*In situ*)	38.55	0.05	0.62	0.175
(Beryllium)	(Lab)	36.87	0.05	0.59	0.175
Moro soil	(*In situ*)	74.18	0.09	1.18	0.315
(Gold)	(Lab)	28.92	0.04	0.46	0.140
(UNSCEAR, 2000)		**59.00**	**0.07**	**≤1**	**0.29**

**Table 12:** Radiation hazard indices for the sampled waters and guinea corn grains.

Locations	AED_ing_ (mSvy^−1^)	ELCR (× 10^−3^)
Ifelodun water (beryllium)	0.15	0.53
Moro water (gold)	0.39	1.35
Ifelodun grain (beryllium)	0.05	0.17
Moro grain (gold)	0.05	0.19
(UNSCEAR, 2000)	1.00	3.75

**Figure 3:**
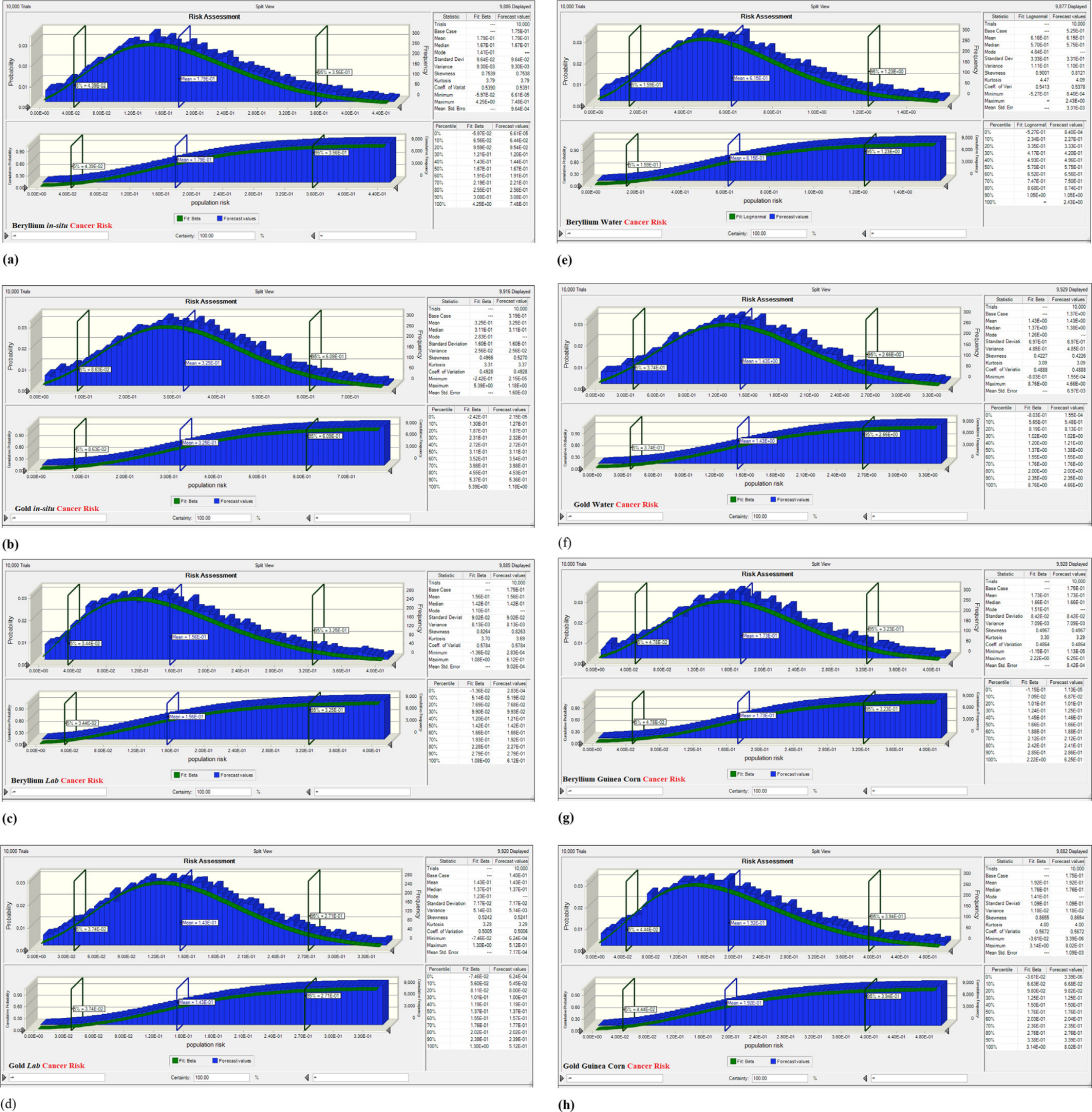
MCS of the Excess Lifetime Cancer Risk associated with the measured primordial radioelements in soil, water and guinea corn grains from the mining sites. (a) Beryllium *in-situ* cancer risk, (b) Gold *in-situ* cancer risk, (c) Beryllium soil cancer risk, (d) Gold soil cancer risk, (e) Beryllium water cancer risk, (f) Gold water cancer risk, (g) Beryllium guinea corn cancer risk, and (h) Gold guinea corn cancer risk.

Table 13 and Figure 3a–h shows the results of the Monte Carlo simulation (*MCS*) for all the mining sites. The average, P 5% and P 95% cumulative probabilities for the cancer risk in all mining fields falls within the recommended limits provided by UNSCEAR (UNSCEAR, 2000). The results also reveal that residents around the Gold mine are the most vulnerable and drinking water route poses greater risk.

**Table 13:** Result of the MCS.

*ELCR* (× 10^−3^)			
	5%	Mean	95%
Beryllium *in-situ*	0.0439	0.1790	0.3560
Gold *in-situ*	0.0863	0.3250	0.6090
Beryllium Soil (Lab)	0.0344	0.1560	0.3250
Gold Soil (Lab)	0.0374	0.1430	0.2710
Beryllium Water	0.1590	0.6150	1.2300
Gold Water	0.3740	1.4300	2.6600
Beryllium Grain	0.0478	0.1730	0.3230
Gold Grain	0.0444	0.1920	0.3940

## 4 Conclusion

The activity concentrations of ^40^K, ^238^U and ^232^Th embedded in guinea corn grains, water and the soil around Beryllium and Gold mining sites in Kwara State, Nigeria, were assessed in this work. The results of the activities revealed that the mining sites are enriched with ^40^K compared to ^238^U and ^232^Th. All the measured activity concentrations of ^40^K, ^238^U and ^232^Th in the soils are lower than the corresponding *in situ* measurements. This could be attributed to the contribution from other terrestrial materials on-site. While the values for the bioaccumulation factor for ^238^U and ^232^Th exceeds the mean value of 0.0062 for ^238^U and 0.0021 for ^232^Th for cereals grains provided by IAEA handbook of parameter values for the prediction of radionuclide transfer factors, the values reported in this study for ^40^K are well below the 0.74 for cereals grains. The outcome of the radiation impact assessment using the Monte Carlo simulations discloses values that are by and large within the recommendation limits of the United Nations Scientific Committee on the Effects of Atomic Radiation (UNSCEAR. Thus, both the mining zones have negligible risk of exposure to ionizing radiation as they are all within the satisfactory limits.

## Declarations

### Author contribution statement

Muyiwa Michael Orosun: Conceived and designed the experiments; Performed the experiments; Analyzed and interpreted the data; Contributed reagents, materials, analysis tools or data; Wrote the paper.

Mojisola Rachael Usikalu; Kayode John OYEWUMI; Gbonjubola Victoria AWOLOLA; Mark Tibbett: Contributed reagents, materials, analysis tools or data.

Maxwell OMEJE; Oluwaseun AJIBOLA: Performed the experiments; Analyzed and interpreted the data.

### Funding statement

Dr Muyiwa Michael Orosun was supported by TETFund IBR [TETFUND/DESS/UNI/ILORIN/2017/RP/VOL.I].

### Data availability statement

Data will be made available on request.

### Competing interest statement

The authors declare no conflict of interest.

### Additional information

No additional information is available for this paper.
